# The Omega-3 Polyunsaturated Fatty Acid DHA Induces Simultaneous Apoptosis and Autophagy via Mitochondrial ROS-Mediated Akt-mTOR Signaling in Prostate Cancer Cells Expressing Mutant p53

**DOI:** 10.1155/2013/568671

**Published:** 2013-06-10

**Authors:** Soyeon Shin, Kaipeng Jing, Soyeon Jeong, Nayeong Kim, Kyoung-Sub Song, Jun-Young Heo, Ji-Hoon Park, Kang-Sik Seo, Jeongsu Han, Jong-Il Park, Gi-Ryang Kweon, Seung-Kiel Park, Tong Wu, Byung-Doo Hwang, Kyu Lim

**Affiliations:** ^1^Department of Biochemistry, College of Medicine, Chungnam National University, Daejeon 301-747, Republic of Korea; ^2^Infection Signaling Network Research Center, Chungnam National University, Daejeon 301-747, Republic of Korea; ^3^Department of Pathology and Laboratory Medicine, Tulane University School of Medicine, New Orleans, LA 70112, USA; ^4^Cancer Research Institute, Chungnam National University, Daejeon 301-747, Republic of Korea

## Abstract

Docosahexaenoic acid (DHA) induces autophagy-associated apoptotic cell death in wild-type p53 cancer cells via regulation of p53. The present study investigated the effects of DHA on PC3 and DU145 prostate cancer cell lines harboring mutant p53. Results show that, in addition to apoptosis, DHA increased the expression levels of lipidated form LC3B and potently stimulated the autophagic flux, suggesting that DHA induces both autophagy and apoptosis in cancer cells expressing mutant p53. DHA led to the generation of mitochondrial reactive oxygen species (ROS), as shown by the mitochondrial ROS-specific probe mitoSOX. Similarly, pretreatment with the antioxidant N-acetyl-cysteine (NAC) markedly inhibited both the autophagy and the apoptosis triggered by DHA, indicating that mitochondrial ROS mediate the cytotoxicity of DHA in mutant p53 cells. Further, DHA reduced the levels of phospho-Akt and phospho-mTOR in a concentration-dependent manner, while NAC almost completely blocked that effect. Collectively, these findings present a novel mechanism of ROS-regulated apoptosis and autophagy that involves Akt-mTOR signaling in prostate cancer cells with mutant p53 exposed to DHA.

## 1. Introduction

Prostate cancer is the second leading cause of male cancer-related death in the USA [1], and migration-associated changes in risk have provided evidence that genetic and environmental factors, such as p53 alteration and dietary fat, contribute to the disease [2–4]. Epidemiological data suggest that while high intake of saturated fatty acids is positively associated with prostate cancer risk, certain omega-3 polyunsaturated fatty acids (*ω*3-PUFAs), in particular docosahexaenoic acid (DHA), seem to prevent this type of cancer [5, 6]. The protective role of *ω*3-PUFAs against prostate cancer is also supported by evidence from in vitro and in vivo studies, and multiple mechanisms have been proposed to account for the association of *ω*3-PUFAs with reduced prostate cancer risk, including (a) modulation of phospholipase A_2_ and oxygenases and synthesis of their corresponding metabolites [7–11], (b) alteration in cell membrane phospholipid composition and receptor function [11–15], and (c) regulation of gene expression and signal transduction [16, 17].

Autophagy and apoptosis are self-destructive processes that share many key regulators, such as reactive oxygen species (ROS). Physiological levels of ROS lead to growth adaption and survival; however, excessive ROS cause irreversible cellular damage, thus provoking autophagy and/or apoptosis [18, 19]. Likewise, many pathways are commonly used by these two processes in response to a single stress. For example, inactivation of Akt-mammalian target of rapamycin (mTOR) signaling is responsible for the parallel occurrence of autophagy and apoptosis in human brain tumor cells exposed to hydrogen peroxide [20].

A recent study shows that ethanolamine derivatives of *ω*3-PUFAs reduce cell viability by inducing autophagy in MCF-7 breast cancer cells [21], which express wild-type p53. We previously reported that autophagy is involved in DHA-induced apoptosis via p53-mediated mTOR signaling in a number of cancer cells harboring wild-type p53 [22]. These observations indicate that the antitumor activity of *ω*3-PUFAs is tightly linked to their ability to trigger autophagy and apoptosis and set the stage for an effective treatment of tumors possessing functional p53; however, since p53 is frequently mutated in human cancers, it remains unclear whether simultaneous induction of autophagy and apoptosis is a universal *ω*3-PUFA antitumor mechanism regardless of p53 status.

To address this issue, we investigated the involvement of autophagy and apoptosis in DHA-treated prostate cancer cell lines with altered p53 status. The results indicate that DHA simultaneously induces autophagy and apoptosis in cancer cells expressing mutant p53 via mitochondrial ROS overproduction and that Akt-mTOR pathway inactivation mediated by ROS may play a key role in DHA-induced cytotoxicity in prostate cancer cells.

## 2. Materials and Methods

### 2.1. Cell Lines, Culture Conditions, and Treatment with Reagents

The human PC3 and DU145 metastatic adenocarcinoma cell lines were purchased from the American Type Culture Collection (Manassas, VA) and grown in RPMI-1640 medium (Invitrogen, Gaithersburg, MD) supplemented with 10% heat-inactivated fetal bovine serum (Invitrogen, Carlsbad, CA), penicillin, and streptomycin at 37°C in a humidified 5% CO_2_ incubator. When required, cells were allowed to grow to 60−70% confluence and were exposed to serum-free medium for 24 h before receiving any treatment.

Absolute ethanol was used to dissolve DHA, eicosapentaenoic acid (EPA), and arachidonic acid (AA) (Cayman Chemical, Michigan) and served as a control treatment (CN). The DNA staining dyes propidium iodide (PI) and 4,6-diamidino-2-phenylindole (DAPI) were obtained from Roche Applied Science (Indianapolis, IN). Unless indicated, all other reagents were from Sigma-Aldrich (ST Louis, MO). The lysosomal inhibitor chloroquine (CQ) and the ROS scavenger N-acetyl-L-cystein (NAC) were dissolved in H_2_O. For exposure to DHA plus CQ or NAC, cells were pretreated with 2 *μ*M CQ or 5 mM NAC for 1 h and then exposed to DHA.

### 2.2. Cell Viability Assay

The cytotoxicity of DHA (alone or in the presence of NAC) was measured using 3-(4,5-dimethylthiazol-2-yl)-2,5-diphenyltetrazolium bromide (MTT). PC3 and DU145 prostate cancer cells were seeded at 1 × 10^4^ cells per well in 96-well plates. At the end of the assay, MTT was added to each well (final concentration 200 *μ*g/mL) and the cultures were incubated at 37°C for 1 h. The supernatants were then removed and the formazan crystals were dissolved by adding dimethyl sulfoxide (200 *μ*L). The absorbance was measured at 570 nm in a microplate spectrophotometer (Thermo Fisher Scientific), and cell viability was expressed as ratios versus untreated CN cells.

### 2.3. Measurement of ROS Production

PC3 and DU145 cell lines seeded in 6-well plates were exposed to DHA in the presence or absence of NAC for 2 h. The ROS probes dihydroethidium (DHE, 5 *μ*M, Molecular Probes, Carlsbad, CA) or MitoSOX (2.5 *μ*M, Molecular Probes, Carlsbad, CA) were then added and the cultures were further incubated at 37°C for 20 min. Direct imaging of ROS in probe-loaded cells was performed by fluorescence microscopy. Alternatively, fluorescence intensity was measured by flow cytometry. At least 10,000 events were analyzed using a FACScan instrument (FACS-Calibur, BD Biosciences, CA).

### 2.4. Analysis of Apoptotic Parameters

Apoptosis was assessed by terminal deoxynucleotidyl transferase dUTP nick end labeling (TUNEL) assay and flow cytometry, as described previously [22].

### 2.5. Plasmid and Transfection

Microtubule-associated protein 1 light chain 3 beta (LC3B) fused to green fluorescent protein (GFP) (kind gift of Dr. Tamotsu Yoshimori, National Institute of Genetics, Mishima, Japan) was transfected using Lipofectamine LTX with Plus reagent (Invitrogen, Carlsbad, CA) as recommended by the vendor. After 24 h of transfection, cells were exposed to 50 *μ*M DHA for 2 h. The transfected cells were then observed under a fluorescence microscope (Olympus iX70, Japan), and fluorescence imaging was conducted to detect the punctate pattern of GFP-LC3B.

### 2.6. Western Blot Analysis

Cells were harvested, washed once with ice-cold phosphate buffered saline, and subjected to Western blot analysis, as described previously [23]. The antibodies were as follows: Akt (#9272), phospho-Akt (S473, #4060), mTOR (#2972), phospho-mTOR (S2448, #2971), and LC3B (#3868) were obtained from Cell Signaling Technology; phospho-Akt (T308, sc-16646), poly (ADP-ribose) polymerase (PARP, H-250, sc-8007), and actin (I-19, sc-1616) were obtained from Santa Cruz Biotechnology. Goat anti-rabbit (401315) and anti-mouse (401215) secondary antibodies were purchased from Calbiochem.

### 2.7. Statistical Analysis

Results are expressed as means ± SD. Significance was assessed by Student's *t* test. *P* < 0.05 was considered statistically significant (*P < 0.05).

## 3. Results and Discussion

### 3.1. DHA Exerts Cytotoxic Effects on Prostate Cancer Cell Lines with Altered p53

**Figure 1:**
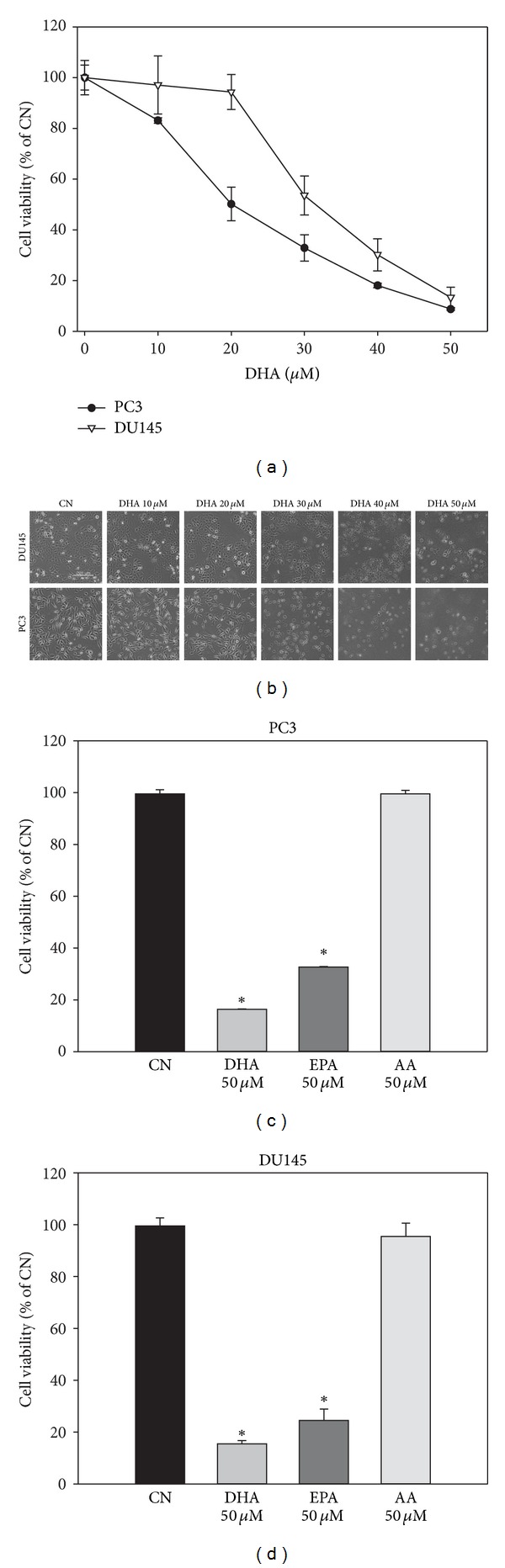
DHA induces cytotoxicity in PC3 and DU145 prostate cancer cells. (a) Reduced cell viability in response to DHA. PC3 and DU145 cells were exposed to 0−50 *μ*M DHA for 24 h and cell viability was measured. (b) Representative images of PC3 (bottom) and DU145 (top) cells exposed to DHA for 24 h (scale bar, 200 *μ*m). (c), (d) Different effects of *ω*3- and *ω*6-PUFAs on the viability of PC3 and DU145 cells. PC3 cells (c) and DU145 cells (d) were exposed to 50 *μ*M DHA, EPA, and AA for 24 h and cell viability was measured by MTT. Data are displayed as means ± SD (*n* > 5; *P < 0.05).

Since the *ω*3-PUFA DHA kills cancer cells with wild-type 53 [22], two commonly used human prostate cancer cell lines with altered p53 status, PC3 (p53-null) and DU145 (P223L/V274F) [24], were used to determine whether the anticancer effect of DHA is dependent on functional p53. DHA decreased PC3 and DU145 cell viability in a concentration-dependent manner, and the DHA concentration of 30 *μ*M suppressed viability of DU145 and PC3 cells by about 50% and 70%, respectively, in the MTT assay (Figure 1(a)), suggesting that DHA is cytotoxic to cancer cells with mutant p53 and that p53 function is not essential to DHA anticancer activity. This observation is consistent with a previous study by Kato et al. [25] showing that the inhibitory effect of DHA on colon cancer growth in vitro and in vivo is not extremely dependent on functional p53.

Morphological analysis of PC3 and DU145 cells demonstrated that DHA induces extensive cell death as evidenced by cell rounding, detachment, and shrinkage (Figure 1(b)). Further, at the same concentrations, another *ω*3-PUFA, EPA, had a lesser cytotoxic effect on PC3 cells and DU145 cells than DHA, while the *ω*6-PUFA AA had no effect or decreased cell growth only slightly (Figures 1(c) and 1(d)). These observations indicate that DHA may exert cytotoxicity mainly by induction of cell death, and that the effects observed in mutant p53 prostate cancer cells might be restricted to *ω*3-PUFAs.

### 3.2. DHA Simultaneously Induces Apoptosis and Autophagy in Prostate Cancer Cells with Altered p53

**Figure 2:**
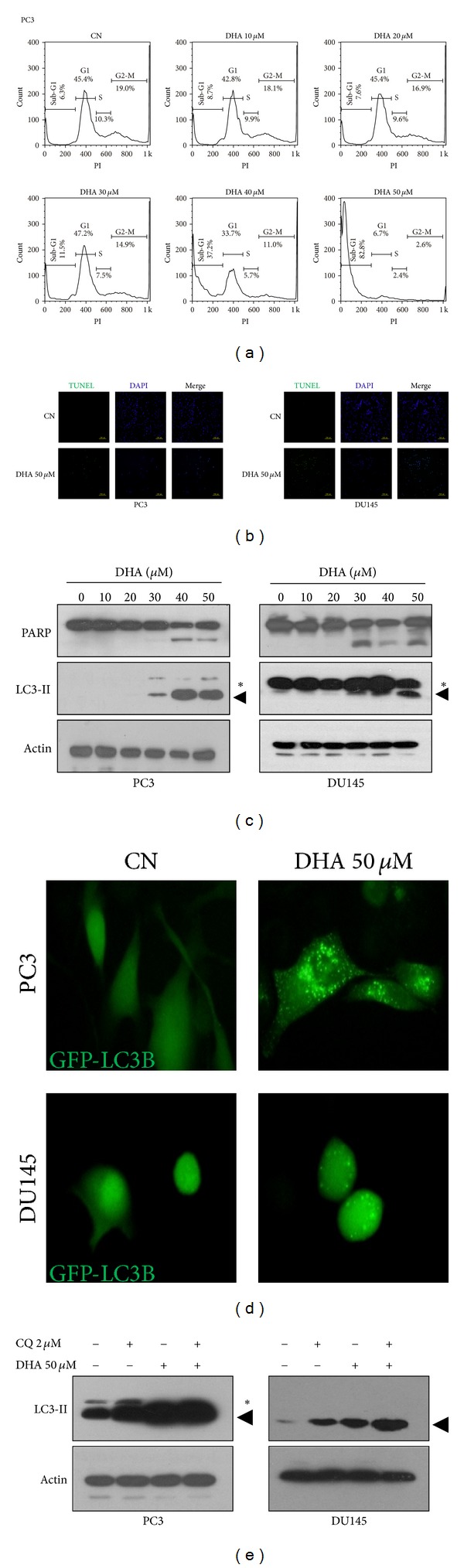
DHA induces apoptosis and autophagic activation in PC3 and DU145 prostate cancer cells. (a) Cell cycle analysis of PC3 cells exposed to DHA. PC3 cells were incubated with 0−50 *μ*M DHA for 24 h and then subjected to flow cytometry after staining with PI. (b) Increased number of TUNEL-positive cells in DHA-treated cells. PC3 (left) and DU145 (right) cells were incubated with 50 *μ*M DHA for 24 h before staining with TUNEL reagents (scale bar, 200 *μ*m). (c) Levels of cleaved PARP and expression levels of LC3-II in response to DHA. PC3 (left) and DU145 (right) cells were incubated with 0−50 *μ*M DHA for 24 h, and PARP cleavage and the expression levels of LC3-II were monitored by Western blot analysis. (d) Representative images of PC3 (top) and DU145 (bottom) cells transiently expressing GFP-LC3B upon exposure to 50 *μ*M DHA for 2 h (magnification, 600x). (e) Effect of DHA on autophagic flux. PC3 (left) and DU145 (right) cells were incubated with 50 *μ*M DHA and/or 2 *μ*M CQ for 24 h and were then subjected to Western blotting for the detection of LC3-II. Black triangle indicates the lipidated form LC3B (LC3-II), and asterisk indicates nonlipidated form of LC3B (LC3-I).

To determine whether, besides cell death, growth arrest may mediate the effect of DHA in prostate cancer cells with mutant p53, cell cycle analysis was performed using PC3 cells exposed to the same concentrations of DHA as those used in the cell viability assays (Figure 2(a)). DHA did not cause G1, S, or G2-M phase cell cycle arrest; instead, it remarkably increased the number of cells with Sub-G1 DNA content, which represents hypodiploid nuclei, a typical characteristic of apoptotic cells [26]. These results provide evidence that DHA reduces the viability of p53-mutant prostate cancer cells by inducing cell death and indicate that apoptosis is involved in that cell death process. Next, we performed experiments to detect TUNEL-positive cells (DNA nicks) and PARP cleavage, two commonly used markers of apoptosis [26], in DHA-treated cells. Results showed marked increases in the number of TUNEL-positive cells (Figure 2(b)) and in the levels of cleaved PARP (Figure 2(c)) in both PC3 and DU145 cells exposed to DHA, confirming that DHA induces apoptosis in prostate cancer cells with altered p53 status.

These findings are consistent with previous studies demonstrating that apoptosis is a common response to DHA in prostate and other tumor cell lines [8, 14, 27–29]. The apoptosis machinery consists of the intrinsic (mitochondria-mediated) and extrinsic (death receptor-mediated) pathways [26], and DHA has been shown to induce apoptosis by both pathways through various cell surface receptors [30–32] and intracellular signaling molecules [33], including the well-documented apoptotic inducer p53 [34]; however, since p53 is mutated in PC3 and DU145 cells, DHA-induced apoptosis in these cells must occur through a mechanism other than p53 activation.

As apoptosis and autophagy are highly interconnected [26] and because the ability of DHA to induce apoptosis is related to its ability to stimulate autophagic activation in tumor cells with wild-type p53 [22], we investigated whether autophagy may also be stimulated by DHA in cell models carrying altered p53. To this end, we first examined the level of lipidated form LC3B (LC3-II), a marker of autophagic membranes [35, 36], in PC3 and DU145 cells exposed to DHA. Western blot analysis showed that DHA increased the expression levels of LC3-II in both cell lines (Figure 2(c)), indicating that DHA increases the amount of autophagic membranes. This observation was confirmed in cells transfected with GFP-LC3B, which showed a higher number of GFP-LC3B puncta than control cells (Figure 2(d)) upon exposure to DHA. Moreover, autophagic flux assays in cells pretreated with CQ, an inhibitor of lysosomal acidification [37], revealed that DHA further increased the expression levels of LC3-II (Figure 2(e)). These observations clearly demonstrate that DHA also stimulates autophagy in prostate cancer cells with mutant p53 and indicate that functional p53 is not essential to DHA-induced autophagy.

Our results so far suggest that, in addition to apoptosis, autophagy is induced by DHA, and that apoptosis and autophagy may act together to mediate DHA cytotoxicity in prostate cancer cells with altered p53. As an evolutionarily conserved process, basal levels of autophagy maintain cellular homeostasis, while overactivated autophagy causes either cell damage by degrading essential cellular components or cell survival by conferring resistance to stress [35, 37]. As previous studies conducted by us and others show that the autophagic activation induced by DHA and its derivatives contributes to the death of cancer cells with wild-type p53 [21, 22], it seems reasonable to speculate that the autophagy induced by DHA might be prodeath in cells expressing mutant p53 as well. Investigation is currently underway to address this issue. 

### 3.3. DHA-Induced Apoptosis and Autophagy Are Mediated by Mitochondrial ROS

**Figure 3:**
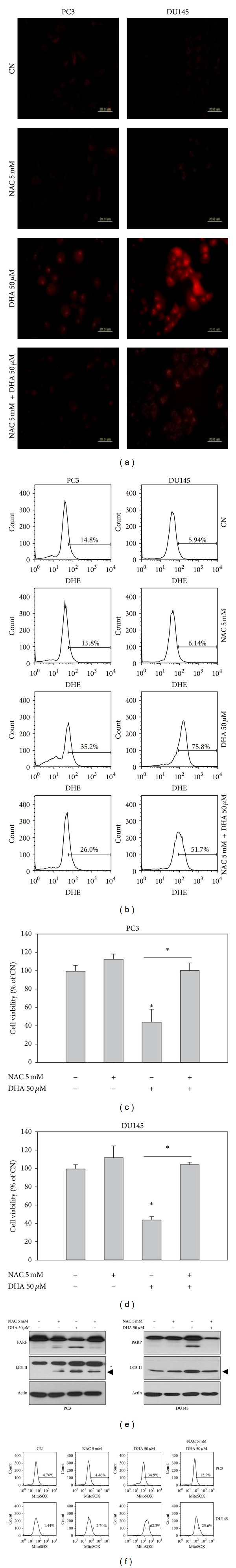
Mitochondrial ROS overproduction mediates DHA-induced apoptosis and autophagy. (a), (b) Increased levels of intracellular ROS induced by DHA in PC3 and DU145 cells. DHE-loaded PC3 and DU145 cells were examined for intracellular ROS accumulation by microscopy (a) and flow cytometry (b) after incubating with 50 *μ*M DHA in the absence or presence of 5 mM NAC for 2 h (scale bar, 200 *μ*m). (c), (d) Effect of the ROS scavenger NAC on the reduction in cell viability induced by DHA. PC3 (c) and DU145 (d) cells were exposed to 50 *μ*M DHA and/or 5 mM NAC for 12 h and cell viability was measured by MTT. Data are displayed as means ± SD. (*n* > 5; *P < 0.05). (e) Effect of NAC on the levels of cleaved PARP and on the expression levels of LC3-II in DHA-treated cells. PC3 (left) and DU145 (right) cells were exposed to 50 *μ*M DHA and/or 5 mM NAC for 12 h, and PARP cleavage and the expression levels of LC3-II were examined by Western blot analysis. Black triangle indicates the lipidated form LC3B (LC3-II), and asterisk indicates nonlipidated form of LC3B (LC3-I). (f) Increased levels of mitochondrial ROS induced by DHA. MitoSOX-loaded PC3 (top) and DU145 (bottom) cells were examined for mitochondrial ROS levels by flow cytometry after incubating with 50 *μ*M DHA in the absence or presence of 5 mM NAC for 2 h.

Multiple lines of evidence indicate that ROS accumulation induced by DHA is responsible for the proapoptotic effect of DHA in tumor cells [33], and ROS have also been implicated in autophagic activation [19, 20]. This knowledge led us to ask whether the simultaneous induction of apoptosis and autophagy by DHA in our cell systems was mediated by ROS. PC3 and DU145 cells were exposed to DHA and the level of intracellular ROS was monitored using the oxidation-sensitive fluorescent probe DHE. DHA resulted in a substantial increase in intracellular ROS accumulation, whereas the antioxidant NAC effectively inhibited that effect in both cell lines, as shown by fluorescence microscopy and flow cytometry (Figures 3(a) and 3(b)). More importantly, NAC pretreatment completely blocked the DHA-induced reduction in cell viability (Figures 3(c) and 3(d)) and reduced the elevated PARP cleavage and LC3-II levels caused by DHA (Figure 3(e)). On the basis of these findings, we conclude that ROS are involved in DHA-induced cytotoxicity in PC3 and DU145 prostate cancer cells and that DHA induces autophagy and apoptosis by triggering intracellular ROS accumulation in these cells.

Moreover, since the major source of intracellular ROS is mitochondria [38], we further examined whether the observed DHA-induced intracellular ROS accumulation originated from mitochondria. As shown in Figure 3(f), in both PC3 and DU145 cells, DHA dramatically increased the signal of the mitochondrial ROS-specific dye MitoSOX, whereas NAC repressed that effect, supporting the mitochondrial origin of DHA-induced ROS in our experimental conditions. Altogether, these data indicate that DHA induces autophagy and apoptosis by triggering mitochondrial ROS overproduction. Although it is not clear how DHA induces mitochondrial ROS in PC3 and DU145 cells, DHA supplementation has been shown to result in its extensive incorporation into mitochondrial membrane phospholipids and to enhance mitochondrial lipid oxidation and ROS generation, leading to mitochondrial dysfunction [39, 40]. It is therefore highly likely that the mitochondrial ROS overproduction observed in PC3 and DU145 cells may result from the direct accumulation of DHA in cell mitochondrial membranes; further study is required to test this hypothesis.

Together, these results are in line with those of others [33, 39, 40], supporting that ROS induction contributes to the antitumor activity of *ω*3-PUFAs; however, it is noteworthy that the effect of *ω*3-PUFAs on ROS has been shown to be inconsistent. Schmidt et al. [41] recently reported that supplements containing EPA and DHA enhance the transcriptional levels of genes related to antioxidative enzymes in both normal and dyslipidemic subjects, indicating an inhibitory effect of *ω*3-PUFAs on oxidative stress. Further, human aortic endothelial cells exposed to EPA and DHA also show reduced ROS production [42]. The inconsistency between augmentation and reduction in oxidative stress caused by *ω*3-PUFAs is not fully understood, but it might be associated with different cellular milieu in which they react with oxidants [43]. 

### 3.4. ROS-Mediated Akt-mTOR Signaling Inactivation Participates in the Autophagy and Apoptosis Induced by DHA

**Figure 4:**
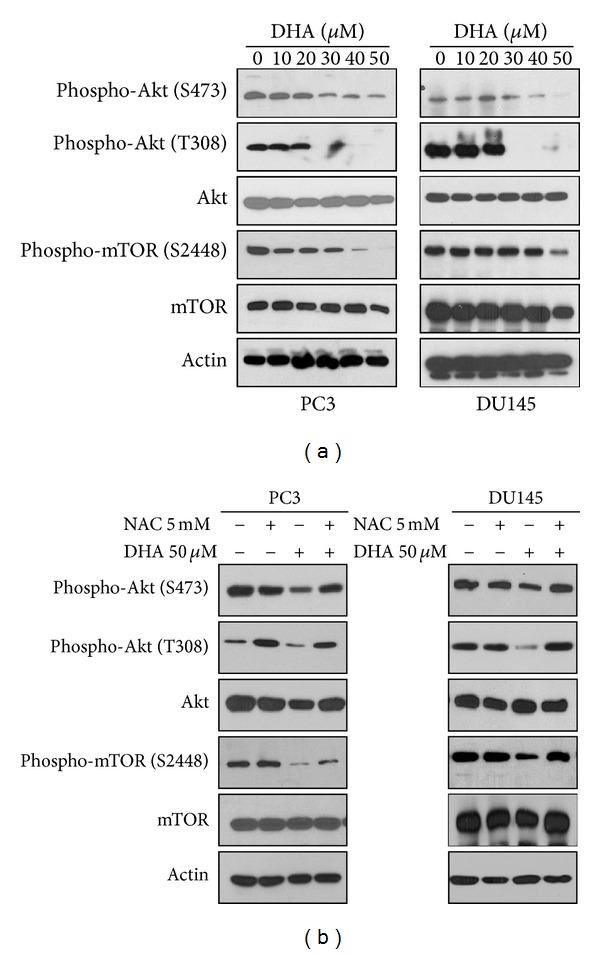
Inhibition of Akt-mTOR signaling contributes to the ROS-mediated apoptosis and autophagy. (a) Inhibition of Akt-mTOR signaling induced by DHA. PC3 (left) and DU145 (right) cells were incubated with 0−50 *μ*M DHA for 24 h, and then the expression levels of Akt-mTOR pathway molecules were detected by Western blot analysis. (b) Effect of NAC on the expression levels of Akt-mTOR pathway molecules in DHA-treated cells. PC3 (left) and DU145 (right) cells were exposed to 50 *μ*M DHA and/or 5 mM NAC for 12 h, and the expression levels of Akt-mTOR pathway molecules were examined by Western blot analysis.

The finding that ROS regulates the simultaneous induction of autophagy and apoptosis by DHA prompted us to investigate the molecular mechanism underlying ROS-induced cytotoxicity. The Akt-mTOR pathway plays a crucial role in prostate cancer development [44], and inhibition of this signaling by ROS is associated with both autophagic activation and apoptosis in cancer cells [20]. Therefore, we hypothesized that Akt-mTOR signaling inactivation might be the mechanism by which DHA induces ROS-mediated autophagy and apoptosis. When PC3 and DU145 cells were exposed to increasing concentrations of DHA, the levels of phosphorylated Akt and mTOR decreased in a concentration-dependent manner whereas the levels of total Akt and mTOR remained unchanged (Figure 4(a)), implying Akt-mTOR signaling inactivation. Next, to obtain direct evidence for the interconnection between Akt-mTOR signaling inhibition and ROS accumulation, PC3 and DU145 cells were preincubated with or without NAC and then exposed to DHA. Western blot analysis revealed that the levels of phosphorylated Akt and mTOR were restored by NAC (Figure 4(b)). These data indicate that the ROS-mediated Akt-mTOR signaling inactivation may be responsible for the autophagy and apoptosis induced by DHA in prostate cancer cells expressing altered p53.

## 4. Conclusions

**Figure 5:**
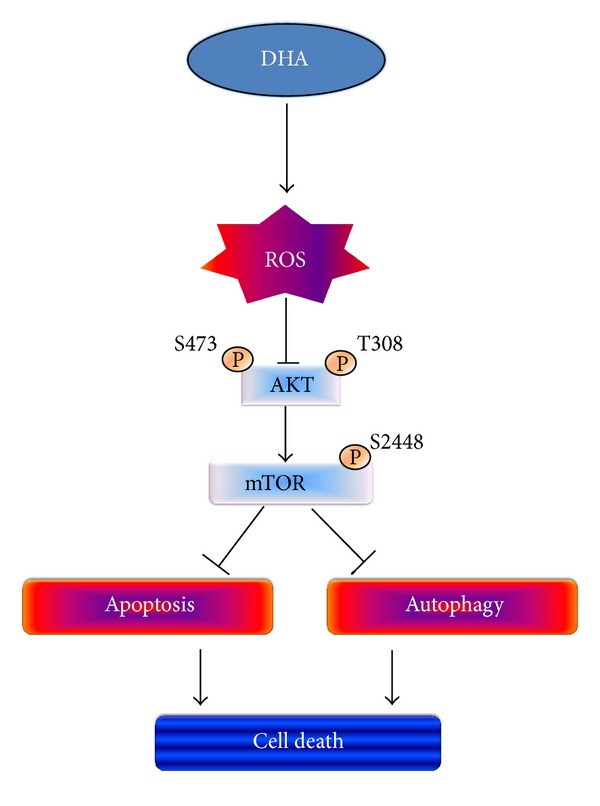
Proposed mechanism of DHA-induced apoptosis and autophagy in prostate cancer cells with altered p53 status.

In summary, the present study shows that the *ω*3-PUFA DHA simultaneously induces autophagy and apoptosis in p53-mutant PC3 and DU145 cells by triggering mitochondrial ROS generation and that inhibition of Akt-mTOR signaling is involved in this process (Figure 5). These findings suggest that DHA may be beneficial to patients with p53-mutant prostate cancer and provide a strong rationale for the use of DHA in the treatment of prostate cancer.
